# 2-(Naphthalen-1-yl)-4-(naphthalen-1-yl­methyl­idene)-1,3-oxazol-5(4*H*)-one

**DOI:** 10.1107/S1600536811015340

**Published:** 2011-04-29

**Authors:** Cevher Gündoğdu, Serap Alp, Yavuz Ergün, Barış Tercan, Tuncer Hökelek

**Affiliations:** a Dokuz Eylül University, Faculty of Arts and Sciences, Department of Chemistry, Tınaztepe, 35160 Buca, Izmir, Turkey; b Karabük University, Department of Physics, 78050, Karabük, Turkey; c Hacettepe University, Department of Physics, 06800 Beytepe, Ankara, Turkey

## Abstract

In the title compound, C_24_H_15_NO_2_, the oxazole ring is oriented at dihedral angles of 10.09 (4) and 6.04 (4)° with respect to the mean planes of the naphthalene ring systems, while the two naphthalene ring systems make a dihedral angle of 4.32 (3)°. Intra­molecular C—H⋯N hydrogen bonds link the oxazole N atom to the naphthalene ring systems. In the crystal, inter­molecular weak C—H⋯O hydrogen bonds link the mol­ecules into centrosymmetric dimers. π–π contacts between the oxazole and naphthalene rings and between the naphthalene ring systems [centroid–centroid distances = 3.5947 (9) and 3.7981 (9) Å] may further stabilize the crystal structure. Three weak C—H⋯π inter­actions also occur.

## Related literature

For the roles of oxazolones in the syntheses of amino acids, peptides, anti­microbial or anti­tumor compounds, immunomodulators, heterocyclic precursors for biosensors coupling and/or photosensitive composition devices for proteins, see: Gottwald & Seebach (1999 ▶); Meiwes *et al.* (1997 ▶); Martinez *et al.* (1964 ▶); Gelmi *et al.* (1997 ▶); Croce *et al.* (1994 ▶); Cannella *et al.* (1996 ▶); Kojima *et al.* (1998 ▶). For applications of the 5-oxazolones, including their use in semiconductor devices because of their promising photophysical and photochemical activity, see: Gündoğdu *et al.* (2010 ▶). For bond-length data, see: Allen *et al.* (1987 ▶).
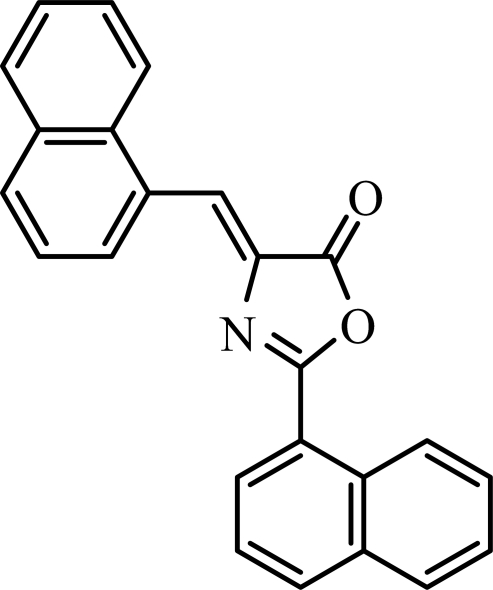


## Experimental

### 

#### Crystal data

C_24_H_15_NO_2_
*M* *_r_* = 349.37
Monoclinic, 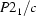

*a* = 18.6927 (5) Å
*b* = 6.0646 (2) Å
*c* = 15.6262 (5) Å
β = 107.212 (2)°
*V* = 1692.11 (9) Å^3^
*Z* = 4
Mo *K*α radiation
μ = 0.09 mm^−1^
*T* = 100 K
0.42 × 0.35 × 0.16 mm

#### Data collection

Bruker Kappa APEXII CCD area-detector diffractometer
Absorption correction: multi-scan (*SADABS*; Bruker, 2005 ▶) *T*_min_ = 0.964, *T*_max_ = 0.986
29257 measured reflections
4260 independent reflections
2911 reflections with *I* > 2σ(*I*)
*R*_int_ = 0.061

#### Refinement

*R*[*F* ^2^ > 2σ(*F* ^2^)] = 0.044
*wR*(*F* ^2^) = 0.136
*S* = 1.07
4260 reflections
244 parameters
H-atom parameters constrained
Δρ_max_ = 0.26 e Å^−3^
Δρ_min_ = −0.25 e Å^−3^

### 

Data collection: *APEX2* (Bruker, 2007 ▶); cell refinement: *SAINT* (Bruker, 2007 ▶); data reduction: *SAINT*; program(s) used to solve structure: *SHELXS97* (Sheldrick, 2008 ▶); program(s) used to refine structure: *SHELXL97* (Sheldrick, 2008 ▶); molecular graphics: *ORTEP-3 for Windows* (Farrugia, 1997 ▶) and *PLATON* (Spek, 2009 ▶); software used to prepare material for publication: *WinGX* (Farrugia, 1999 ▶) and *PLATON*.

## Supplementary Material

Crystal structure: contains datablocks I, global. DOI: 10.1107/S1600536811015340/xu5196sup1.cif

Structure factors: contains datablocks I. DOI: 10.1107/S1600536811015340/xu5196Isup2.hkl

Supplementary material file. DOI: 10.1107/S1600536811015340/xu5196Isup3.cml

Additional supplementary materials:  crystallographic information; 3D view; checkCIF report

## Figures and Tables

**Table 1:** Hydrogen-bond geometry (Å, °) *Cg*1, *Cg*2 and *Cg*4 are the centroids of the C1—C3/C8—C10, C3—C8 and C15—C19/C24 rings, respectively.

*D*—H⋯*A*	*D*—H	H⋯*A*	*D*⋯*A*	*D*—H⋯*A*
C2—H2⋯N1	0.95	2.34	3.0110 (19)	127
C10—H10⋯O2^i^	0.95	2.46	3.324 (2)	152
C11—H11⋯O2^i^	0.95	2.47	3.3601 (18)	155
C23—H23⋯N1	0.95	2.25	2.908 (2)	126
C9—H9⋯*Cg*4^ii^	0.95	2.87	3.543 (2)	129
C18—H18⋯*Cg*1^iii^	0.95	2.61	3.381 (2)	139
C20—H20⋯*Cg*2^iii^	0.95	2.75	3.450 (2)	131

Symmetry codes: (i) 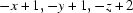; (ii) 
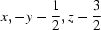
; (iii) 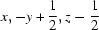.

**Fig. 1.**
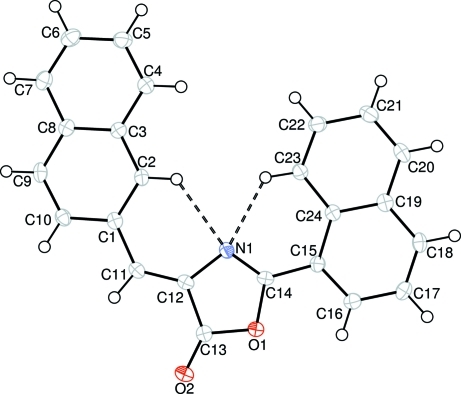
The molecular structure of the title molecule with the atom-numbering scheme. Displacement ellipsoids are drawn at the 50% probability level. The intra-molecular C-H···N bonds are shown as dashed lines.

**Fig. 2.**
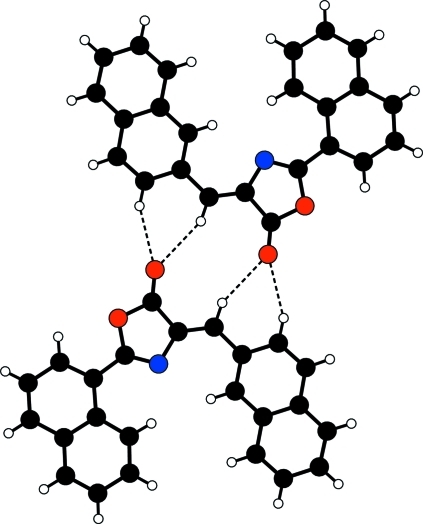
A view of the crystal packing of the title compound. The C-H···O hydrogen bonds are shown as dashed lines.

## supplementary crystallographic information

### Comment

Oxazolones that are internal anhydrides of acyl amino acids are important class of five-membered heterocycles. They are highly versatile intermediates used for the syntheses of several organic molecules, including amino acids, peptides (Gottwald & Seebach, 1999; Meiwes *et al.*, 1997), antimicrobial or antitumor compounds (Martinez *et al.*, 1964; Gelmi *et al.*, 1997), immunomodulators, heterocyclic precursors for biosensors coupling (Croce *et al.*, 1994; Cannella *et al.*, 1996) and/or photosensitive composition devices for proteins (Kojima *et al.*, 1998). They can be easily prepared from N-acyl amino acids by dehydration. 5-Oxazolones have also a wide range of applications including their use in semiconductor devices because of their promising photophysical and photochemical activities (Gündoğdu *et al.*, 2010). The present study was undertaken to ascertain the crystal structure of the title compound.

The title compound consists of an oxazol ring and two naphthalene groups (Fig. 1), where the bond lengths are close to standard values (Allen *et al.*, 1987). The intramolecular C-H···N hydrogen bonds link the oxazol nitrogen atoms to the naphthalene groups (Table 1 and Fig. 1).

An examination of the deviations from the least-squares planes through individual rings shows that rings A (C1—C3/C8—C10), B (C3—C8), C (O1/N1/C12—C14), D (C15—C19/C24) and E (C19—C24) are planar. The naphthalene groups, containing the rings A, B and D, E are also nearly planar [with maximum deviations of 0.022 (2) Å for atom C6 and -0.061 (2) Å for atom C17] with dihedral angles of A/B = 1.62 (3) and D/E = 3.58 (4) °. Ring C is oriented with respect to the planar naphthalene groups at dihedral angles of 10.09 (4) and 6.04 (4) °, respectively, while the two naphthalene groups are oriented at a dihedral angle of 4.32 (3)°.

In the crystal, intermolecular weak C—H···O hydrogen bonds link the molecules into centrosymmetric dimers (Table 1 and Fig. 2). The π–π contacts between the oxazol and naphthalene rings and between the naphthalene groups Cg3—Cg4^i^ and Cg1—Cg5^i^ [symmetry code: (i) x, y - 1, z, where Cg1, Cg3, Cg4 and Cg5 are centroids of the rings A (C1—C3/C8—C10), C (O1/N1/C12—C14), D (C15—C19/C24) and E (C19—C24), respectively, may further stabilize the structure, with centroid-centroid distances of 3.5947 (9) and 3.7981 (9) Å, respectively. There also exist three weak C-H···π interactions (Table 1).

### Experimental

For the preparation of the title compound, (I), α-naphtaldehyde (0.74 g, 5 mmol), naphthalen-1-yl glycine (1.14 g, 5 mmol), acetic anhydride (2.49 ml, 12 mmol) and sodium acetate (0.41 g, 5 mmol) were heated until the mixture just liquefied, and then heating was continued for a further 2 h at 353 K. After completion of the reaction, ethanol (25 ml) was added and the mixture was kept at room temperature for 18 h. The solid product obtained was purified by washing with cold ethanol, hot water and a small amount of hexane, respectively. It was crystallized from hot ethanol (yield; 0.22 g, 30%, m.p. 453 K).

### Refinement

H-atoms were positioned geometrically with C—H = 0.95 Å, and constrained to ride on their parent atoms with *U*_iso_(H) = 1.2*U*_eq_(C).

### Crystal data

**Table:**

C_24_H_15_NO_2_	*F*(000) = 728
*M**_r_* = 349.37	*D*_x_ = 1.371 Mg m^−^^3^
Monoclinic, *P*2_1_/*c*	Mo *K*α radiation, λ = 0.71073 Å
Hall symbol: -P 2ybc	Cell parameters from 3844 reflections
*a* = 18.6927 (5) Å	θ = 2.3–25.8°
*b* = 6.0646 (2) Å	µ = 0.09 mm^−^^1^
*c* = 15.6262 (5) Å	*T* = 100 K
β = 107.212 (2)°	Block, yellow
*V* = 1692.11 (9) Å^3^	0.42 × 0.35 × 0.16 mm
*Z* = 4	

### Data collection

**Table:**

Bruker Kappa APEXII CCD area-detector diffractometer	4260 independent reflections
Radiation source: fine-focus sealed tube	2911 reflections with *I* > 2σ(*I*)
graphite	*R*_int_ = 0.061
φ and ω scans	θ_max_ = 28.5°, θ_min_ = 1.1°
Absorption correction: multi-scan (*SADABS*; Bruker, 2005)	*h* = −25→25
*T*_min_ = 0.964, *T*_max_ = 0.986	*k* = −8→8
29257 measured reflections	*l* = −20→20

### Refinement

**Table:**

Refinement on *F*^2^	Primary atom site location: structure-invariant direct methods
Least-squares matrix: full	Secondary atom site location: difference Fourier map
*R*[*F*^2^ > 2σ(*F*^2^)] = 0.044	Hydrogen site location: inferred from neighbouring sites
*wR*(*F*^2^) = 0.136	H-atom parameters constrained
*S* = 1.07	*w* = 1/[σ^2^(*F*_o_^2^) + (0.0712*P*)^2^ + 0.094*P*] where *P* = (*F*_o_^2^ + 2*F*_c_^2^)/3
4260 reflections	(Δ/σ)_max_ = 0.001
244 parameters	Δρ_max_ = 0.26 e Å^−^^3^
0 restraints	Δρ_min_ = −0.25 e Å^−^^3^

### Special details

**Table:**

Geometry. All esds (except the esd in the dihedral angle between two l.s. planes) are estimated using the full covariance matrix. The cell esds are taken into account individually in the estimation of esds in distances, angles and torsion angles; correlations between esds in cell parameters are only used when they are defined by crystal symmetry. An approximate (isotropic) treatment of cell esds is used for estimating esds involving l.s. planes.
Refinement. Refinement of F^2^ against ALL reflections. The weighted R-factor wR and goodness of fit S are based on F^2^, conventional R-factors R are based on F, with F set to zero for negative F^2^. The threshold expression of F^2^ > 2sigma(F^2^) is used only for calculating R-factors(gt) etc. and is not relevant to the choice of reflections for refinement. R-factors based on F^2^ are statistically about twice as large as those based on F, and R- factors based on ALL data will be even larger.

### Fractional atomic coordinates and isotropic or equivalent isotropic displacement parameters (Å^2^)

**Table:**

	*x*	*y*	*z*	*U*_iso_*/*U*_eq_	
O1	0.44739 (6)	−0.05303 (17)	1.11266 (7)	0.0226 (3)	
O2	0.51969 (6)	0.2213 (2)	1.08529 (8)	0.0339 (3)	
N1	0.33689 (7)	0.0161 (2)	1.00801 (8)	0.0182 (3)	
C1	0.30409 (8)	0.4225 (2)	0.88040 (10)	0.0183 (3)	
C2	0.24089 (8)	0.2914 (2)	0.85707 (10)	0.0183 (3)	
H2	0.2421	0.1521	0.8852	0.022*	
C3	0.17453 (8)	0.3596 (2)	0.79239 (10)	0.0185 (3)	
C4	0.11021 (9)	0.2236 (3)	0.76755 (11)	0.0231 (4)	
H4	0.1109	0.0848	0.7960	0.028*	
C5	0.04717 (9)	0.2900 (3)	0.70303 (11)	0.0263 (4)	
H5	0.0044	0.1969	0.6866	0.032*	
C6	0.04523 (9)	0.4962 (3)	0.66073 (11)	0.0265 (4)	
H6	0.0012	0.5407	0.6156	0.032*	
C7	0.10607 (9)	0.6326 (3)	0.68407 (10)	0.0230 (4)	
H7	0.1038	0.7716	0.6554	0.028*	
C8	0.17232 (8)	0.5692 (2)	0.75047 (10)	0.0188 (3)	
C9	0.23747 (9)	0.7022 (2)	0.77577 (10)	0.0208 (3)	
H9	0.2368	0.8431	0.7490	0.025*	
C10	0.30132 (9)	0.6317 (2)	0.83806 (10)	0.0200 (3)	
H10	0.3444	0.7234	0.8533	0.024*	
C11	0.37403 (8)	0.3572 (2)	0.94585 (10)	0.0197 (3)	
H11	0.4156	0.4516	0.9508	0.024*	
C12	0.38719 (8)	0.1798 (2)	1.00029 (10)	0.0191 (3)	
C13	0.45933 (9)	0.1340 (3)	1.06678 (11)	0.0227 (4)	
C14	0.37283 (8)	−0.1109 (2)	1.07250 (10)	0.0175 (3)	
C15	0.34675 (8)	−0.3067 (2)	1.10825 (10)	0.0179 (3)	
C16	0.39709 (9)	−0.4187 (3)	1.17708 (10)	0.0217 (3)	
H16	0.4460	−0.3605	1.2022	0.026*	
C17	0.37753 (9)	−0.6171 (3)	1.21081 (10)	0.0229 (4)	
H17	0.4126	−0.6898	1.2592	0.027*	
C18	0.30808 (9)	−0.7050 (2)	1.17389 (10)	0.0212 (3)	
H18	0.2961	−0.8434	1.1947	0.025*	
C19	0.25329 (9)	−0.5932 (2)	1.10494 (10)	0.0192 (3)	
C20	0.18039 (9)	−0.6812 (3)	1.07013 (10)	0.0227 (4)	
H20	0.1690	−0.8202	1.0909	0.027*	
C21	0.12628 (9)	−0.5699 (3)	1.00735 (11)	0.0254 (4)	
H21	0.0775	−0.6308	0.9846	0.031*	
C22	0.14316 (9)	−0.3641 (3)	0.97648 (11)	0.0238 (4)	
H22	0.1052	−0.2854	0.9334	0.029*	
C23	0.21331 (8)	−0.2757 (3)	1.00745 (10)	0.0202 (3)	
H23	0.2234	−0.1371	0.9852	0.024*	
C24	0.27132 (8)	−0.3873 (2)	1.07232 (10)	0.0172 (3)	

### Atomic displacement parameters (Å^2^)

**Table:**

	*U*^11^	*U*^22^	*U*^33^	*U*^12^	*U*^13^	*U*^23^
O1	0.0174 (5)	0.0225 (6)	0.0250 (6)	−0.0026 (4)	0.0017 (5)	0.0044 (5)
O2	0.0192 (6)	0.0353 (7)	0.0414 (8)	−0.0083 (5)	0.0002 (5)	0.0108 (6)
N1	0.0193 (7)	0.0164 (6)	0.0190 (6)	−0.0017 (5)	0.0059 (5)	0.0001 (5)
C1	0.0221 (8)	0.0179 (7)	0.0165 (7)	−0.0010 (6)	0.0081 (6)	−0.0024 (6)
C2	0.0216 (8)	0.0164 (7)	0.0176 (8)	−0.0013 (6)	0.0070 (6)	0.0008 (6)
C3	0.0188 (8)	0.0200 (8)	0.0172 (8)	0.0012 (6)	0.0060 (6)	−0.0004 (6)
C4	0.0209 (8)	0.0236 (8)	0.0245 (8)	−0.0014 (6)	0.0064 (7)	0.0029 (7)
C5	0.0193 (8)	0.0328 (9)	0.0255 (9)	−0.0023 (7)	0.0048 (7)	0.0016 (7)
C6	0.0219 (8)	0.0315 (9)	0.0252 (9)	0.0069 (7)	0.0054 (7)	0.0023 (7)
C7	0.0260 (9)	0.0223 (8)	0.0220 (8)	0.0052 (6)	0.0091 (7)	0.0021 (6)
C8	0.0223 (8)	0.0197 (7)	0.0157 (7)	0.0018 (6)	0.0074 (6)	−0.0013 (6)
C9	0.0305 (9)	0.0147 (7)	0.0195 (8)	−0.0001 (6)	0.0110 (7)	0.0008 (6)
C10	0.0227 (8)	0.0190 (8)	0.0195 (8)	−0.0035 (6)	0.0079 (6)	−0.0023 (6)
C11	0.0195 (8)	0.0196 (7)	0.0206 (8)	−0.0041 (6)	0.0072 (6)	−0.0019 (6)
C12	0.0168 (7)	0.0205 (8)	0.0199 (8)	−0.0030 (6)	0.0053 (6)	−0.0025 (6)
C13	0.0212 (8)	0.0211 (8)	0.0248 (8)	−0.0012 (6)	0.0052 (7)	0.0028 (7)
C14	0.0147 (7)	0.0197 (7)	0.0179 (8)	−0.0009 (6)	0.0042 (6)	−0.0028 (6)
C15	0.0199 (8)	0.0186 (7)	0.0159 (7)	0.0002 (6)	0.0064 (6)	0.0001 (6)
C16	0.0198 (8)	0.0238 (8)	0.0207 (8)	0.0004 (6)	0.0047 (6)	0.0001 (6)
C17	0.0266 (9)	0.0230 (8)	0.0185 (8)	0.0041 (7)	0.0058 (7)	0.0043 (6)
C18	0.0296 (9)	0.0172 (7)	0.0192 (8)	−0.0008 (6)	0.0110 (7)	0.0017 (6)
C19	0.0242 (8)	0.0186 (7)	0.0169 (8)	−0.0003 (6)	0.0095 (6)	−0.0031 (6)
C20	0.0279 (9)	0.0201 (8)	0.0232 (8)	−0.0072 (6)	0.0123 (7)	−0.0029 (6)
C21	0.0224 (8)	0.0286 (9)	0.0255 (9)	−0.0075 (7)	0.0073 (7)	−0.0023 (7)
C22	0.0193 (8)	0.0283 (9)	0.0222 (8)	−0.0002 (6)	0.0039 (7)	0.0015 (7)
C23	0.0200 (8)	0.0202 (8)	0.0204 (8)	−0.0018 (6)	0.0061 (6)	0.0010 (6)
C24	0.0202 (8)	0.0181 (7)	0.0146 (7)	−0.0009 (6)	0.0072 (6)	−0.0019 (6)

### Geometric parameters (Å, °)

**Table:**

O1—C13	1.3949 (18)	C11—C1	1.456 (2)
O1—C14	1.3935 (17)	C11—H11	0.9500
O2—C13	1.2015 (18)	C12—C11	1.348 (2)
N1—C12	1.3971 (18)	C12—C13	1.465 (2)
N1—C14	1.2880 (18)	C15—C14	1.456 (2)
C1—C2	1.380 (2)	C15—C16	1.380 (2)
C1—C10	1.424 (2)	C16—H16	0.9500
C2—C3	1.411 (2)	C17—C16	1.404 (2)
C2—H2	0.9500	C17—H17	0.9500
C3—C4	1.414 (2)	C18—C17	1.364 (2)
C3—C8	1.425 (2)	C18—H18	0.9500
C4—C5	1.365 (2)	C19—C18	1.420 (2)
C4—H4	0.9500	C20—C19	1.414 (2)
C5—C6	1.410 (2)	C20—H20	0.9500
C5—H5	0.9500	C21—C20	1.361 (2)
C6—C7	1.366 (2)	C21—C22	1.407 (2)
C6—H6	0.9500	C21—H21	0.9500
C7—H7	0.9500	C22—C23	1.366 (2)
C8—C7	1.413 (2)	C22—H22	0.9500
C9—C8	1.416 (2)	C23—C24	1.418 (2)
C9—H9	0.9500	C23—H23	0.9500
C10—C9	1.367 (2)	C24—C15	1.440 (2)
C10—H10	0.9500	C24—C19	1.426 (2)
			
C14—O1—C13	105.32 (11)	C11—C12—C13	123.81 (14)
C14—N1—C12	106.50 (13)	O1—C13—C12	105.36 (12)
C2—C1—C10	118.66 (14)	O2—C13—O1	121.07 (14)
C2—C1—C11	123.31 (14)	O2—C13—C12	133.56 (15)
C10—C1—C11	118.03 (13)	O1—C14—C15	115.87 (12)
C1—C2—C3	121.52 (14)	N1—C14—O1	114.99 (13)
C1—C2—H2	119.2	N1—C14—C15	129.14 (14)
C3—C2—H2	119.2	C16—C15—C14	118.16 (14)
C2—C3—C4	121.43 (14)	C16—C15—C24	119.98 (14)
C2—C3—C8	119.38 (14)	C24—C15—C14	121.84 (13)
C4—C3—C8	119.18 (14)	C15—C16—C17	121.39 (14)
C3—C4—H4	119.7	C15—C16—H16	119.3
C5—C4—C3	120.55 (15)	C17—C16—H16	119.3
C5—C4—H4	119.7	C16—C17—H17	120.1
C4—C5—C6	120.32 (15)	C18—C17—C16	119.81 (14)
C4—C5—H5	119.8	C18—C17—H17	120.1
C6—C5—H5	119.8	C17—C18—C19	121.13 (14)
C5—C6—H6	119.7	C17—C18—H18	119.4
C7—C6—C5	120.53 (15)	C19—C18—H18	119.4
C7—C6—H6	119.7	C18—C19—C24	119.71 (14)
C6—C7—C8	120.72 (15)	C20—C19—C18	120.54 (14)
C6—C7—H7	119.6	C20—C19—C24	119.73 (14)
C8—C7—H7	119.6	C19—C20—H20	119.5
C7—C8—C3	118.68 (14)	C21—C20—C19	121.06 (15)
C7—C8—C9	122.97 (14)	C21—C20—H20	119.5
C9—C8—C3	118.32 (13)	C20—C21—C22	119.51 (15)
C8—C9—H9	119.4	C20—C21—H21	120.2
C10—C9—C8	121.21 (14)	C22—C21—H21	120.2
C10—C9—H9	119.4	C21—C22—H22	119.5
C1—C10—H10	119.5	C23—C22—C21	121.06 (15)
C9—C10—C1	120.91 (14)	C23—C22—H22	119.5
C9—C10—H10	119.5	C22—C23—C24	121.11 (14)
C1—C11—H11	115.9	C22—C23—H23	119.4
C12—C11—C1	128.18 (14)	C24—C23—H23	119.4
C12—C11—H11	115.9	C19—C24—C15	117.75 (13)
N1—C12—C13	107.81 (13)	C23—C24—C15	124.75 (14)
C11—C12—N1	128.32 (14)	C23—C24—C19	117.50 (14)
			
C14—O1—C13—O2	−178.58 (15)	C13—C12—C11—C1	−177.33 (14)
C14—O1—C13—C12	1.12 (15)	N1—C12—C13—O1	−1.62 (16)
C13—O1—C14—N1	−0.25 (17)	N1—C12—C13—O2	178.04 (18)
C13—O1—C14—C15	179.03 (12)	C11—C12—C13—O1	175.62 (14)
C14—N1—C12—C11	−175.61 (16)	C11—C12—C13—O2	−4.7 (3)
C14—N1—C12—C13	1.47 (16)	C16—C15—C14—O1	−0.7 (2)
C12—N1—C14—O1	−0.79 (17)	C16—C15—C14—N1	178.50 (15)
C12—N1—C14—C15	−179.96 (15)	C24—C15—C14—O1	−179.11 (12)
C10—C1—C2—C3	0.4 (2)	C24—C15—C14—N1	0.1 (2)
C11—C1—C2—C3	−179.11 (13)	C14—C15—C16—C17	−175.58 (14)
C2—C1—C10—C9	0.2 (2)	C24—C15—C16—C17	2.9 (2)
C11—C1—C10—C9	179.72 (14)	C18—C17—C16—C15	1.6 (2)
C1—C2—C3—C4	178.99 (14)	C19—C18—C17—C16	−3.6 (2)
C1—C2—C3—C8	−0.4 (2)	C20—C19—C18—C17	−177.05 (14)
C2—C3—C4—C5	−178.21 (14)	C24—C19—C18—C17	1.2 (2)
C8—C3—C4—C5	1.2 (2)	C21—C20—C19—C18	176.55 (14)
C2—C3—C8—C7	178.33 (14)	C21—C20—C19—C24	−1.7 (2)
C2—C3—C8—C9	−0.1 (2)	C22—C21—C20—C19	0.1 (2)
C4—C3—C8—C7	−1.1 (2)	C20—C21—C22—C23	1.0 (2)
C4—C3—C8—C9	−179.54 (13)	C21—C22—C23—C24	−0.5 (2)
C3—C4—C5—C6	−0.5 (2)	C22—C23—C24—C15	179.71 (13)
C4—C5—C6—C7	−0.4 (2)	C22—C23—C24—C19	−1.0 (2)
C3—C8—C7—C6	0.2 (2)	C19—C24—C15—C14	173.26 (13)
C5—C6—C7—C8	0.5 (2)	C19—C24—C15—C16	−5.2 (2)
C9—C8—C7—C6	178.61 (14)	C23—C24—C15—C14	−7.5 (2)
C10—C9—C8—C3	0.7 (2)	C23—C24—C15—C16	174.11 (14)
C10—C9—C8—C7	−177.68 (14)	C15—C24—C19—C18	3.2 (2)
C1—C10—C9—C8	−0.7 (2)	C15—C24—C19—C20	−178.59 (13)
C12—C11—C1—C2	−8.0 (3)	C23—C24—C19—C18	−176.15 (13)
C12—C11—C1—C10	172.50 (15)	C23—C24—C19—C20	2.1 (2)
N1—C12—C11—C1	−0.7 (3)		

### Hydrogen-bond geometry (Å, °)

**Table:**

Cg1, Cg2 and Cg4 are the centroids of the rings A (C1—C3/C8—C10), B (C3—C8) and D (C15—C19/C24), respectively.

**Table:**

*D*—H···*A*	*D*—H	H···*A*	*D*···*A*	*D*—H···*A*
C2—H2···N1	0.95	2.34	3.0110 (19)	127
C10—H10···O2^i^	0.95	2.46	3.324 (2)	152
C11—H11···O2^i^	0.95	2.47	3.3601 (18)	155
C23—H23···N1	0.95	2.25	2.908 (2)	126
C9—H9···Cg4^ii^	0.95	2.87	3.543 (2)	129
C18—H18···Cg1^iii^	0.95	2.61	3.381 (2)	139
C20—H20···Cg2^iii^	0.95	2.75	3.450 (2)	131

Symmetry codes: (i) −*x*+1, −*y*+1, −*z*+2; (ii) *x*, −*y*−1/2, *z*−3/2; (iii) *x*, −*y*+1/2, *z*−1/2.
